# A pre- and during Pandemic Survey of Sars-Cov-2 Infection in Stray Colony and Shelter Cats from a High Endemic Area of Northern Italy

**DOI:** 10.3390/v13040618

**Published:** 2021-04-03

**Authors:** Eva Spada, Fabrizio Vitale, Federica Bruno, Germano Castelli, Stefano Reale, Roberta Perego, Luciana Baggiani, Daniela Proverbio

**Affiliations:** 1 Laboratorio di Ricerca di Medicina Emotrasfusionale Veterinaria (REvLab), Dipartimento di Medicina Veterinaria (DIMEVET), Università degli Studi di Milano, 26900 Lodi, Italy; luciana.baggiani@unimi.it (L.B.); daniela.proverbio@unimi.it (D.P.); 2 Centro di Referenza Nazionale per le Leishmaniosi (C.Re.Na.L), Istituto Zooprofilattico Sperimentale (IZS) della Sicilia A. Mirri, 90129 Palermo, Italy; fabrizio.vitale@izssicilia.it (F.V.); federica.bruno@izssicilia.it (F.B.); germano.castelli@izssicilia.it (G.C.); stefano.reale@izssicilia.it (S.R.)

**Keywords:** COVID-19, SARS-CoV-2, stray cat, shelter cats, ELISA, RT-PCR, northern Italy

## Abstract

Cats are susceptible to infection with severe acute respiratory syndrome Coronavirus 2 (SARS-CoV-2). Whilst a number of studies have been performed worldwide on owned cats, limited data are available on stray, colony or shelter cats. We investigated SARS-CoV-2 infection in a stray cat population before and during human outbreaks of SARS-CoV-2 in cities in the Lombardy region in northern Italy, a high endemic region for SARS-CoV-2, using serological and molecular methods. A cohort of different samples were collected from 241 cats, including frozen archived serum samples from 136 cats collected before the 2019 coronavirus disease (COVID-19) pandemic and serum, pharyngeal and rectal swab samples from 105 cats collected during the SARS-CoV-2 outbreak. All pre-pandemic samples tested seronegative for antibodies against the nucleocapsid of SARS-CoV-2 using indirect enzyme linked immunosorbent assay (ELISA) test, while one serum sample collected during the pandemic was seropositive. No serological cross-reactivity was detected between SARS-CoV-2 antibodies and antibodies against feline enteric (FECV) and infectious peritonitis coronavirus (FIPC), Feline Immunodeficiency Virus (FIV), Feline Calicivirus (FCV), Feline Herpesvirus-1 (FHV-1), Feline Parvovirus (FPV), *Leishmania infantum*, *Anaplasma phagocytophilum*, *Rickettsia* spp., *Toxoplasma gondii* or *Chlamydophila felis*. No pharyngeal or rectal swab tested positive for SARS-CoV-2 RNA on real time reverse transcription-polymerase chain reaction (rRT-PCR). Our data show that SARS-CoV-2 did infect stray cats in Lombardy during the COVID-19 pandemic, but with lower prevalence than found in owned cats. This should alleviate public concerns about stray cats acting as SARS-CoV-2 carriers.

## 1. Introduction

In December 2019, human cases of coronavirus disease 2019 (COVID- 19), caused by the severe acute respiratory syndrome Coronavirus 2 (SARS-CoV-2), were first reported in Wuhan, China [1]. Since then, a large number of human COVID-19 cases have been reported around the world and a global pandemic was officially declared by the World Health Organization (WHO) [1]. Italy was one of the first countries outside China to be affected and was amongst the most severely affected countries in Europe, with a high number of documented cases and deaths. Despite major efforts to control the COVID-19 outbreak, the disease is still spreading in the Lombardy region in northern Italy, one of the worst affected Italian regions with 583,251 human cases of SARS-COV-2 infection reported to date [2]. The Milan metropolitan area was the first area in Western countries to be severely hit by the spread of SARS-CoV-2, with 200,808 human cases of SARS-COV-2 infection to date [2,3].

As already reported during the 2003 SARS-CoV-1 outbreak [4], cats are susceptible to SARS-CoV-2 infection as demonstrated by experimental studies in which cats exposed to the virus can be infected and are able to transmit the disease via respiratory droplets and by direct contact to other felines [5,6,7,8].

Domestic cats were also sporadically reported to be naturally infected by SARS-CoV-2 [9,10,11,12]. The first naturally infected cats were reported in New York in mid-April 2020 [13]. Since then, other cases have been reported in European countries including the Netherlands [14], France [9], Italy [15] and Spain [16,17]. These reported episodes highlight that while cats can be infected by SARS-CoV-2, they do not necessarily show clinical signs. Clinical signs of SARS-CoV-2 infection in cats are mainly mild upper respiratory disease, including coughing, nasal and ocular discharge, sneezing, mild lethargy, loss of appetite [11,13] and digestive signs such as anorexia, vomiting and cough [9]. Clinical signs of pneumonia were described for the first time in a cat in Italy that died soon after diagnosis. In that case, SARS-CoV-2 may well have been the cause of the cat’s death, but it could also have been an incidental finding [15]. Other reports describe subclinically infected cats, with no evidence of clinical signs or lesions potentially caused by SARS-CoV-2 or with only signs related to concurrent disease [16,17]. This is as reported in most experimental infections where SARS-CoV-2 infection in cats does not cause any obvious signs [5,6,18]. Most previous reports described natural cases in cats related to COVID-19−affected owners or other people that came into contact with them [10,11,12,16,17,19].

Experimentally [5,6,7,8] and naturally infected cats show seroconversion after SARS-CoV-2 infection, [17]. Serological surveys of pet cat populations report infection rates ranging from 0 to 43.8% [11,12,18,20,21] with the high percentage being in cats in contact with COVID-19 infected owners [11,12] for which the risk of testing seropositive was as much as eight times higher for pets sharing a home with a COVID-19 affected person than for pets in homes of unknown status [12]. At present, there is no evidence of SARS-CoV-2 transmission from cats to people.

Whilst a number of studies have been performed on owned cats worldwide in the last year [12,16,17,18,19,20,22,23,24], limited data are available on stray cats, either from colonies or shelter cats and all these studies were performed in China on a limited number of cats [19,22]. In these seropositivity was only detected in shelter cats with a prevalence of 13% (six out of 46 tested cats from three animal shelters) [19].

Stray colony free-ranging and shelter cats are very common in Italy and often have close contact with human populations. These animals are an important sentinel for local disease trends and for the detection of the emergence of new pathogens and infectious diseases, such as that caused by *Leishmania infantum* in cats [25] or for feline vector-borne infections and zoonosis found in stray cats in Northern Italy [26,27]. Active disease surveillance and monitoring of these feline populations provides early disease detection, protecting both the people and the cats from disease outbreaks. In addition to being neutered, cats in the Trap-Neutered-Release (TNR) programs in Italy are usually fed and watered regularly by volunteer caretakers, treated for parasites and infectious diseases and given veterinary care if they become sick or injured. Thus, it is important to know the prevalence of SARS-CoV-2 in these feline populations, especially in COVID-19 outbreak areas, such as northern Italy, in order to identify potential reservoirs and sources of infection that are important for both animal and human health.

In this study, we investigated the serological and molecular presence and prevalence of SARS-CoV-2 infection in a cohort of stray colony and shelter cats living in cities in Lombardy region, northern Italy using serological and molecular techniques, to better understand the epidemiological roles of these cats in SARS-CoV-2 infection.

## 2. Materials and Methods

### 2.1. Sample Population

Samples were collected from a total of 241 cats mainly from two cities (i.e., Milan and Monza Brianza) and surrounding areas, in Lombardy region, northern Italy. Samples were collected pre- and during COVID-19 pandemic, with the cutoff between the two periods defined as the date of first detection of SARS-CoV-2 RNA in the environment—as found in sewage samples from Milan city on 18 December 2019 [28].

From the pre-pandemic period we analyzed 136 frozen archived serum samples, 89 from free-ranging stray colony cats and 47 from abandoned shelter cats, collected before December 2019. These samples had mostly been collected for previously published epidemiological studies, including one on *Leishmania infantum* and co-infections (*Leishmania infantum, Anaplasma phagocytophilum*, *Rickettsia* spp., *Toxoplasma gondii*, *Chlamydophila felis,* Feline Immunodeficiency Virus (FIV) and Feline Leukemia virus (FeLV)) in stray colony cats [25]. Some samples had been used for a serosurvey on the antibodies against Feline Calicivirus (FCV), Feline Herpesvirus-1 (FHCV-1) and Feline Parvovirus (FPV), in stray cats [29]. Some of these serum samples were also known to be from feline enteric (FECV) seropositive cats and from cats infected by infectious peritonitis coronavirus (FIPC).

Pandemic samples were collected until February 2021 from 105 cats, 77 free-ranging stray colony cats and 28 abandoned shelter cats. These consisted of paired serum samples, oro-pharyngeal and rectal swabs from 75 stray colony cats and 23 shelter cats. In seven cats, rectal and oro-pharyngeal swabs were not collected, while in six cats, serum was not collected, giving a total of 99 serum samples available for serological analysis and 98 paired rectal and oro-pharyngeal swabs to be analyzed by molecular methods

Deep oro-pharyngeal swabs were collected by insertion and rotation of sterile swabs into the posterior pharynx and tonsillar areas. Rectal swabs were collected by inserting the swab 1.5 cm past the anal opening and rotating the swab gently. Swab samples were stored at −20 °C and shipped within 24 h to the Experimental Zooprophylactic Institute (IZS) of Sicily, where real time reverse transcription-polymerase chain reaction (rRT-PCR) analysis was performed to determine the presence of SARS-CoV-2 RNA. Whole blood samples (2.5 mL) were drawn from jugular vein into plain collection tubes to obtain serum after centrifugation, which was aliquoted and stored at −20 °C prior to being sent for enzyme-linked immunosorbent assay (ELISA) antibody tests at IZS of Sicily. All samples were collected when the cats were under general anesthesia before performing the neutering surgery during trap, neuter and release (TNR) sterilization programs that were part of a national program to control stray pet populations under Italian National Law (law no. 281/1991).

Full signalment details, including breed, sex, age, clinical status, province in which the cat was trapped and clinical status for each cat (in particular, presence of respiratory signs) were collected when available.

The protocols for the study and animal welfare were reviewed and approved by the Animal Welfare Bioethical Committee of the University of Milan (approval number OPBA _91_2020). Frozen archived serum samples were collected from cats following protocols already approved by Animal Welfare Bioethical Committee of the University of Milan and were collected with the consent of legal representatives of the stray colony or shelter cats.

### 2.2. Enzyme-Linked Immunosorbent Assay (ELISA) Antibody Test

Antibody presence against the nucleocapsid of SARS-CoV-2 antigen was tested by double antigen indirect enzyme linked immunosorbent assay (ID-Screen^®^ ELISA, SARS-CoV-2 Double Antigen Multi-species, IDvet, Grabels, France), following the manufacture’s instruction and as previously used and described [9]. The kit specifically detects anti-SARS-CoV-2 nucleocapsid (N-Protein) antibodies. Briefly, samples to be tested were diluted 1:2 in the sample dilution buffer and incubated for 45 min at 37 °C. After washing, the multi-species conjugate, diluted in conjugate buffer, was added for 30 min at room temperature. The substrate solution (TMB) was added after the final wash and the optical densities (OD) read at 450 nm after addition of stop solution. Positive (PC) and negative controls (NC) were used in the analysis.

For each sample the “Sample/Positive control” (S/P) ratio was calculated and expressed as a percentage (S/P%) (1):

(1) $$S/P\;\%\;=\frac{\mathrm{ODSample}\;-\;\mathrm{ODNC}}{\mathrm{ODPC}\;–\;\mathrm{ODNC}\;}\times 100$$

Based on the manufacturer’s ELISA cut-off, samples with S/P% less than or equal to 50% were considered negative, between 50% and 60% are considered as doubtful. Greater than or equal to 60% were considered as positive.

### 2.3. RNA Extraction and Real Time Reverse Transcription-Polymerase Chain Reaction (rRT-PCR)

The deep oropharyngeal swabs and rectal swabs were kept on cool-packs until arrival in the laboratory. RNA was extracted from clinical samples employing the Mag-Bind^®^ Viral RNA Xpress Kit (Omega Bio-tek, Norcross, GA, USA) according to the manufacturer’s instructions. The RNA was tested for SARS-CoV-2 RNA in assay RT-PCR to check for the presence of E, N2 and RdRp gene sequences (TIB Molbiol Lightmix^®^ Modular Assays, Berlin, Germany). A volume as 5 µL of extracted RNA, was employed in a 20 μL reaction test contained, 10 μL of 2× Brilliant one step RT-PCR reaction buffer (Agilent Germany; containing 0.4 mM of each deoxyribose triphosphates (dNTP) and 3.2 mM magnesium sulphate). Primer and probe sequences were employed at concentrations and conditions optimized, respectively, according to the Centers for Diseases Control (CDC) protocol and Corman paper [30]. The probes E, RP were labelled with FAM and Blackhole, the N2 probe was labelled with VIC and Blackhole). All oligonucleotide primers and probes were synthesized by Biolegio (Nijmegen, the Netherlands). Thermal cycling was performed at 50 °C for 12 min for reverse transcription, followed by 95 °C for 3 min and then 45 cycles of 95 °C for 15 s, 60 °C for 30 s and qPCR reactions were carried out using an Applied Biosystems QuantStudio 3 PCR System (Thermo Fisher Scientific, Waltham, MA, USA). Because the employed primers and probes are able to detect all known sequences of SARS-CoV-2, we did not need external sources of specific 2019-nCoV RNA, although we employed a positive diagnostic sample as positive control in each Real time plate.

## 3. Results

The demographic and clinical data of cats surveyed in the study are summarized in Table 1. 

All 136 sera collected pre-pandemic were seronegative by indirect ELISA.

Out of the 99 sera collected in cats during the pandemic, one (1.0%) was ELISA positive with a S/P% of 200%. Tests were performed in triplicate and repeated three times with similar results and the mean OD of the sample was 1.74 with a standard deviation of 0.085. The three replicates were performed by two operators using different batches of the kit.

Additional analyzes were performed on archived frozen aliquots of this serum sample showing it to be seronegative for retrovirus infection by FIV and FeLV at rapid ELISA kit (SNAP^®^ Combo Plus FeLV Ag/FIV Ab, IDEXX Laboratories, Europe), seronegative at immunofluorescence antibody test (IFAT) for IgG against FCoV and seropositive at IFAT for IgG against *Leishmania infantum*. All available data for this cat are reported in Table 2.

Some pre-pandemic serum samples used in this study had been previously serologically analyzed and tested positive for antibodies to common feline infectious pathogens using ELISA or IFAT technique. None of these samples crossreacted with SARS-CoV-2 nucleocapsid antibodies tested by double antigen ELISA assay used in this study, as shown in Table 3.

All rectal and oro-pharyngeal swabs tested negative for SARS-CoV-2 RNA at rRT-PCR.

## 4. Discussion

In this observational study, we detected a very low presence of SARS-CoV-2 antibodies in stray cats in Lombardy during the COVID-19 pandemic. A total of 105 cats were tested and only one (1%) cat sampled during the pandemic was positive for antibodies against SARS-CoV-2, while none tested positive by RT-PCR. None of the 136 pre-pandemic samples tested positive for SARS-CoV-2 antibodies.

Until now, limited data have been available on SARS-CoV-2 infection in stray cats. To the author’s knowledge, only two studies have investigated the infection in a limited number of stray or shelter cats and both studies were performed in China. No specific antibodies were detected using double-antigen sandwich ELISA in 21 street cats in Wuhan, China [22]. Another study tested 46 abandoned cats from three shelters in the same city between January and March 2020 and found that six out 46 (13.0%) shelter cats were seropositive on ELISA, four of which had neutralization antibodies on virus neutralization test (VNT), with no clinical signs [19]. The difference in SARS-CoV-2 prevalence reported in previous studies may be explained by the different characteristics of investigated populations (owned versus free-ranging stray or shelter cats). However, results may also have been affected by the SARS-CoV-2 epidemic situations in the area investigated, the different types and sensitivity of serological tests used and criteria used for defining seropositivity [12]. Finally, the age of the tested cats may have been a factor, as experimental studies have shown that juvenile cats are more susceptible to the virus and died with severe histological lesions in the nasal and tracheal mucosa epithelium and lungs [5].

Results from this survey of SARS-CoV-2 infection in stray colony and shelter cats living in areas where viral transmission was active in the human population confirms field observations that cats can seroconvert under the normal conditions [11,12,18,24]. However, despite most (98/105, 93.3%) of the cats surveyed during the pandemic coming from the province of Monza and Brianza (MB) a high endemic area for SARS-CoV-2 where a total of 55,482 infectious human cases were found [2], only one cat tested seropositive for SARS-CoV-2 antibodies and no cat tested positive for viral RNA. These results demonstrate that SARS-CoV-2 has infected stray cats in the Lombardy region of northern Italy, but as expected, this occurred with a much lower prevalence than both in people and owned cats [24], in which SARS-CoV-2 infection is a consequence of close contact with a COVID-19 infected owner [11,12].

Owned cats from Lombardy region were previously surveyed [24] using plaque reduction neutralization test and two RT-PCR assays targeting nucleoprotein and envelope protein genes. SARS-CoV-2-neutralizing antibodies were detected in 11/191 cats (5.8%), with titers ranging from 1:20 to 1:1280. As in our study, all cats tested negative for SARS-CoV-2 RNA in rectal, oro-pharyngeal and nasal swabs, suggesting the absence of active SARS-CoV-2 infection in the feline population investigated. These cats should therefore pose no health risk for people or other cats. In addition, the PCR negative results in seropositive cats suggests that although cats can seroconvert, they may shed virus for relatively short periods of time. In experimental studies, cats stopped shedding virus by 10 days post infection (dpi) and developed neutralizing antibody responses by 7–13 dpi [5].

Our serological results suggest that the ELISA positive cat had been exposed to the virus, although viral RNA detection was negative, as reported in previous studies performed in cats in China [18,19,20] and in Italy [24]. We collected and performed molecular analysis on oro-pharyngeal and rectal swabs from colony stray and shelter cats and conducted SARS-CoV-2 specific rtRT-PCR checking for the presence of E, N2 and RdRp gene sequences and found no positive samples. This indicates that all surveyed cats were free of infection at the time of sampling. The main reasons might be that the viral RNA load was too low to be detected or more likely, that the period for which the cat shed SARS-CoV-2 may have been very short [9] and the acute infection period was missed in the study. Under these circumstances, serological diagnosis is more useful since the SARS-CoV-2 specific antibodies in cats can be detected for a long time after infection [22].

Although it is not possible to identify how stray cats become infected with SARS-CoV-2, it is reasonable to speculate that these infections are probably due to contact with a SARS-CoV-2 polluted environment or wastewater contaminated with SARS-CoV-2 RNA as found in Milan city in northern Italy [28]. It is possible that infection was due to contact with COVID-19 patients who take care of and feed the cats, as happened with a tiger at the Wildlife Conservation Society’s Bronx Zoo, New York, suspected to be infected by SARS-CoV-2 positive zookeeper [31]. Our free-ranging stray cat could also have become infected via SARS-CoV-2 contamination as a result of cat-to-cat transmission. Experimental infections of domestic cats demonstrated not only that they are susceptible to SARS-CoV-2 infection, but are also able to spread the virus to co-housed contact animals [5,6,7,8]. In cities in northern Italy is not uncommon for owned cats to have free outdoor access and, in this way, they could encounter stray cats and engage with them in territorial struggles. As the virus is present in the saliva of infected animals, SARS-CoV-2 could be directly transmitted through bites from an owned infected cat during the territorial struggles. In addition, uninfected cats could contract the virus from contaminated feces, as SARS-CoV-2 RNA shows tropism to the digestive tract [5], with the virus replicating in enterocytes and can be detected in the feces of experimentally virus inoculated cats [5] and in naturally infected cats [9], tigers and lions [31].

The first laboratory-confirmed Italian human COVID-19 case was identified in Lombardy on February 20, 2020 [3], however many studies have shown that SARS-CoV-2 infection was already circulating in Milan in 2019. These studies were based on (I) genomic characterization and phylogenetic analysis on complete SARS-CoV-2 genomes first isolated from people [3]; (II) a seroprevalence study conducted on healthy blood donors in the province of Milan, between 12 and 17 February 2020, in which 2.0% (5 out 300) of donors already displayed antibodies against SARS-CoV-2 [32]; (III) a serosurvey study on 184 individuals, who presented for blood donation between December 2019 and March 2020 at the main blood center in Milan city, in which the first IgM positive test against SARS-CoV-2 was detected on December 9th 2019 [33]; (IV) SARS-CoV-2 RBD-specific antibodies detected in 111 out 959 (11.6%) individuals, from September 2019 (14%) in Milan province, Lombardy [34]. In addition, a study that analyzed 40 composite influent wastewater samples collected between October 2019 and February 2020 in cities of regions in northern Italy including Milan, found 15 positive samples for SARS-CoV-2 RNA with the earliest dates back to 18 December 2019 [28]. Serum samples collected in our study before the outbreak of SARS-CoV-2 were found to be serologically negative and this documents for the first time that no antibodies were present in the stray feline population before the human COVID-19 outbreak in northern Italy, as already demonstrated in cats surveyed in China [19,20] and in France [12]. 

Both enteric (FECV) and FIP (FIPV) coronavirus seropositive sera showed no cross-reactivity with the SARS-CoV-2 serological test used in this study, as previously shown in studies using the receptor-binding domain (RBD) protein ELISA [19,21], IFAT [21], VNT [24] and microsphere immunoassay [12]. This sero-prevalence study was therefore not affected by feline coronavirus infection, which is endemic in stray cats in Lombardy region of northern Italy, with a seroprevalence of 39% in 82 stray cats captured from courtyards in urban areas of Milan in 2014 [35]. In addition, we tested sera already known to be seropositive for many other protozoal, bacterial and viral infections caused by *Leishmania infantum*, *Anaplasma phagocytophilum*, *Rickettsia* spp, *Toxoplasma gondii*, *Chlamydophila felis*, FIV, FCV, FHCV-1 and FPV and found no cross reactivity with SARS-CoV-2 ELISA in any of these samples. Collectively these results show that the ELISA test used in our study has good specificity without cross-reaction with common pathogens or feline coronaviruses present in free-ranging or abandoned stray un-owned feline populations.

Previous case reports have raised the question of whether SARS-CoV-2 infection may exacerbate existing disorders in cats [15] or, conversely, if preexisting comorbidities could predispose cats to become infected with SARS-CoV-2. The SARS-CoV-2 seropositive stray colony cat found in our study was asymptomatic and additional tests showed that he was seronegative for FIV and FeLV retrovirus infections, as shown in cats in previous reports [15,17]. The cat was also seronegative for FCoV, but had antibodies against *Leishmania infantum*, a protozoal infection that is sporadically found in stray cat populations in Lombardy region in northern Italy and, in particular, in stray cats from Milan city where the incidence has been increasing in epidemiological surveys since 2010 [25,35,36].

Our study has some limitations. Surveillance systems relying on a single serological test could result in antigenic cross-reactivity and diagnostic errors; therefore, as a rule, two different tests should be used for confirmation of SARS-CoV-2 serological results. We tested the seropositive sample thrice using three different kits of the same ELISA test and this resulted in the same OD, confirming that no diagnostic errors occurred in analyzing this sample. In addition, we tested serum samples seropositive for different bacterial, protozoal and virus pathogens and found no cross-reaction with SARS-CoV-2 ELISA which was negative in all these samples. However, we did not additionally investigate our samples by other serological methods such as IFAT or VNT, in order to confirm the antibody status and assess the level of neutralizing antibodies in the ELISA seropositive cat. In a previous study the IFAT confirmed the antibody-positive status of the ELISA pre-screened serum samples, but neutralizing antibodies were only detected in 33.3% (2/6) of the ELISA- and IFAT-positive serum samples and titers of the VNT test did not correspond to the OD values of the ELISA test [21]. This has also been observed in other studies [12,19] and is most likely due to a delayed production of neutralizing antibodies as shown in studies in ferrets in which the neutralizing antibodies appear with a delay of around two weeks after the detection of antibodies by IFAT [37]. In addition, an antibody progression study on two cats showed that the detection of neutralizing antibodies is relatively transient and decreases over time [19]. Therefore, a neutralization assay, although being a gold standard assay, has its own limitations. In addition, VNT is not widely available and it is more difficult to perform than other serological tests, in particular when applied to numerous sample populations. 

Results from ELISA tests in human samples are influenced by the type of antigen, with whole spike protein or receptor binding domain of spike antigens giving better specificity and sensitivity when compared to whole virus, membrane or nucleocapsid proteins [38]. A recent human study [39] showed that antibody responses against viral spike and nucleocapsid proteins were equally sensitive in the acute phase of infection, but that responses against nucleocapsid appear to wane in the post-infection phase whereas those against the spike protein persist. The most sensitive serological assay in both acute and post-infection phases seems, therefore, to be those using the spike protein as the binding antigen. The same study [39] demonstrated that population-based seroprevalence studies using only the anti-nucleocapsid assays underestimate the seroprevalence by 30 to 45% when compared to anti-spike antibody assays. In addition, detection of both IgA and IgG may increase sensitivity in human patients, particularly for people experiencing paucisymptomatic or asymptomatic infection [40]. Based on this information, another important limitation of our study is that it used only the nucleocapsid protein as the antigen for serology. As previously highlighted in another similar veterinary study [12], this may have resulted in an underestimation of our serological prevalence data and the infection risk of COVID-19 in our population could be much higher. However, at the time we started our serological survey, no multispecies commercial ELISA kits based on whole spike protein or the receptor binding domain of spike antigens were available nor were tests for both IgG and IgA. 

Another limitation relates to the characteristics of the investigated feline population. Anamnestic and clinical data on free-ranging stray and shelter cats are inaccurate and this limited our understanding of the potential source of SARS-CoV-2 infection in the seropositive cat. We report that SARS-CoV-2 can be transmitted to stray cats, but we cannot know if this happened through contact with people carrying COVID-19 or from environmental contamination or contact with other infected cats.

## 5. Conclusions

This study provides serological evidence for limited SARS-CoV-2 infection in stray cats in Lombardy region of northern Italy, one of the worst affected Italian regions. The negative molecular results showed that these cats are free of infections and the very low seroprevalence indicates that free-ranging stray or shelter cats play a limited role in transmission of SARS-CoV-2 during the pandemic. Collectively, these results showed that there is no indication of SARS-CoV-2 circulating in stray cats. As recently reported for owned cats [41], at this time, the consensus remains that there is no evidence that stray cats are a source of infection for people or other pets and this should relieve the public concerns that stray cats can act as SARS-CoV-2 carriers.

## Figures and Tables

**Table 1:** Data on stray colony and shelter feline populations living in northern Italy investigated for presence and prevalence of SARS-CoV-2 infection using enzyme-linked immunosorbent assay (ELISA) and real time reverse transcription-polymerase chain reaction (rRT-PCR).

Variable		Total *n* = 241	Pre-Pandemic *n* = 136	During Pandemic *n* = 105
Age (years) mean ± SD (range)		2.3 ± 2.6 (0.2–15)	2.5 ± 2.7 (0.4–12)	2.1 ± 2.6 (0.2–15)
Age (years)	Young (≤1)	83 (34.4%)	40 (16.6%)	43 (17.8%)
	Adult (1–10)	133 (55.2%)	76 (31.5%)	57 (23.7%)
	Senior (>10)	6 (2.5%)	5 (2.1%)	1 (0.4%)
	Unknown	19 (7.9%)	15 (6.2%)	4 (1.7%)
Breed	Domestic shorthair	236 (97.9%)	134 (55.6%)	102 (42.3%)
	Mixed Chartreux	2 (0.8%)	2 (0.8%)	0 (0.0%)
	Mixed Siamese	3 (1.2%)	0 (0.0%)	3 (1.2%)
Gender	Male	100 (41.5%)	55 (22.8%)	45 (18.7%)
	Female	138 (57.3%)	78 (32.4%)	60 (24.9%)
	Unknown	3 (1.2%)	3 (1.2%)	0 (0.0%)
Province	Monza Brianza (MB)	157 (65.1%)	59 (24.5%)	98 (40.7%)
	Milano (MI)	83 (34.4%)	76 (31.5%)	7 (2.9%)
	Lodi (LO)	1 (0.4%)	1 (0.4%)	0 (0.0%)
Clinical status	Unhealthy	94 (39.0%)	70 (29.0%)	24 (10.0%)
	Respiratory signs	19 (7.9%)	10 (4.1%)	9 (3.7%)
	Non-respiratory signs	75 (31.1%)	60 (24.9%)	15 (6.2%)
	Healthy	139 (57.7%)	59 (24.5%)	80 (33.2%)
	Unknown	8 (3.3%)	7 (2.9%)	1 (0.4%)

**Table 2:** Demographic, clinical and serological data for a stray colony cat testing seropositive for antibodies against nucleocapsid protein of SARS-Cov-2, by ELISA.

Origin	Stray Colony Cat (Province of Monza and Brianza)
Gender	Male (intact)
Breed	DSH
Age	3 years
Reason for evaluation	Orchidectomy
Clinical status	Healthy
ELISA SARS-CoV-2	200%
rRT-PCR SARS-CoV-2 (pharyngeal and rectal sample)	Negative
IFAT FCoV (cut-off 1:100)	Negative
ELISA for antibodies against FIV	Negative
ELISA for FeLV antigens	Negative
IFAT for *L. infantum* (cut-off: 1:80)	Positive 1:160

DSH: domestic shorthair; ELISA: enzyme-linked immunosorbent SARS-CoV-2: assay; severe acute respiratory syndrome coronavirus 2; OD: optical density; rRT-PCR: real time reverse transcription-polymerase chain reaction; IFAT: immunofluorescence antibody test; FIV: feline immunodeficiency virus; FeLV: feline leukemia virus.

**Table 3:** Number of seropositive samples and relative antibody titers against different feline pathogens that tested negative at double antigen indirect enzyme linked immunosorbent assay (ELISA) for antibodies against nucleocapsid of SARS-CoV-2 in a population of 241 stray cats in northern Italy.

Pathogen	Test	*n* Seropositive Samples	Antibody Titer Range
*Leishmania infantum*	IFAT	22	1:40 to 1:160
*Anaplasma phagocytophilum*	IFAT	2	1:64 to 1:256
*Rickettsia* spp.	IFAT	2	1:128
*Toxoplasma gondii* (IgG)	IFAT	20	1:32 to > 1:256
*Chlamydophila felis*	IFAT	32	1:40 to > 1:320
Feline enteric coronavirus (FECV)	ELISA	4	121 to 133 U
Feline infectious peritonitis virus (FIPV)	IFAT	3	1:800 to ≥ 1:3200
Feline Immunodeficiency Virus (FIV)	ELISA	14	nd
Feline Parvovirus (FPV)	ELISA	8	1:80 to >1:640
Feline Herpesvirus 1 (FHV-1)	ELISA	10	1:16 to 1:64
Feline Calicivirus (FCV)	ELISA	14	1:64 to >1:256

ELISA: enzyme linked immunosorbent assay; IFAT: immunofluorescence antibody test. nd: not determined.

## Data Availability

All study data are included in the article.
